# Visceral Leishmaniasis after Anti-Interleukin 17A (IL-17A) Therapy in a Patient Affected by Psoriatic Arthritis

**DOI:** 10.3390/tropicalmed7100319

**Published:** 2022-10-20

**Authors:** Tommaso Lupia, Silvia Corcione, Valentina Fornari, Barbara Rizzello, Roberta Bosio, Maria Teresa Brusa, Francesco Giuseppe De Rosa

**Affiliations:** 1 Unit of Infectious Diseases, Cardinal Massaia, 14100 Asti, Italy; 2 Department of Medical Sciences, Infectious Diseases, University of Turin, 10126 Turin, Italy; 3 School of Medicine, Tufts University, Boston, MA 02111, USA

**Keywords:** *Leishmania*, psoriasis, arthritis, interleukin-17, secukinumab

## Abstract

The reactivation of latent *Leishmania* infection in chronic diseases and immunocompromised hosts is a broad and heterogeneous field in medicine and infectious diseases. We reported one of the first cases of Visceral Leishmaniasis occurring in a Caucasian middle-aged man living in an endemic country (Italy) for *Leishmania infantum* infection following secukinumab treatment for psoriatic arthritis. The patient was cured with a Liposomal Amphotericin B (L-AmB, 3 mg/Kg on days 1–5, followed by a dose on days 10, 17, 24, 31 and 38) regimen, after which his anti-interleukin 17 treatment was restarted—without recurrence in the follow-up.

## 1. Introduction

Interleukin-17 (IL-17) is a proinflammatory cytokine that plays a critical role in the human immune response. It is a crucial factor in the pathogenesis of many autoimmune diseases, mainly psoriasis and psoriatic arthritis (PA) [1].

Despite it being thought for several years to be produced by Th17 cells, better understanding of cellular sources of IL-17 in psoriasis and PA has recently led to the conclusion that macrophages, dendritic cells, mast cells, γδ T cells, αβ T cells and innate lymphoid cells are also involved in its release [2].

The IL-17 group includes five members, IL-17A to F, and is part of the IL-23/IL-17 immunologic pathway, which is responsible for initiating and feeding the progression of the whole disease pattern. By up-regulating downstream gene transcription, this cytokine axis, in synergy with TNF-α, not only promotes differentiation, activation, proliferation and survival of Th17 cells, but also directly targets skin and joint tissues, triggering inflammation, coagulation, and bone/joint damage [3,4].

Among its various activities, IL-17 can also increase the expression of antimicrobial factors (i.e., lipocalin 2, S100A, beta-defensins) and psoriasis autoantigen that promotes the production of proinflammatory cytokines, empowering the proinflammatory cascade [5,6]. Therapies targeting IL-17 (secukinumab, ixekizumab, brodalumab) are broadly used as a biological treatment of psoriasis and PA, but its role in mediating immune response against infections has yet to be understood. Probably because of its immunologic pattern of disease, leishmaniasis is currently being investigated as a possible factor linked to psoriasis and PA [4,5]. The link between visceral leishmaniasis and IL-7 is with regards to its role in controlling *Leishmania* spp. replication by inducing Th1 response and parasite clearance; indeed, it has been demonstrated that stimulation with *Leishmania* spp. antigens may induce IL-17 and IL-23 production by peripheral-blood mononucleated cells [5,6].

Leishmaniasis is a protozoal infection that might be caused by over 20 Leishmania species, transmitted to humans by infected female phlebotomine sandfly bites. We know about three main forms of leishmaniasis: cutaneous leishmaniasis, mucocutaneous leishmaniasis and VL [7]. Many names can be used to popularly refer to Leishmaniasis i: Aleppo boil, Aleppo button, and Aleppo evil; Baghdad boil; Biskra button and Biskra nodule; Calcutta ulcer; chiclero ulcer; Delhi boil; Jericho button; Kandahar sore; Lahore sore; Oriental button and Oriental sore; Pian bois; Uta for cutaneous leishmania; black fever; dum-dum fever; and Kala-azar for visceral leishmania [8].

It has a worldwide distribution. L. infantum is the most common aetiological agent of VL in Europe and Mediterranean territories and, along with other species, represents a global burden of disease, since endemic areas have become more extended over recent decades. Adaptation phenomena of vectors to urban environments and currently-occurring climate changes contribute to the phlebotomine sandfly’s fitness in new settings, including broader areas in Northern and Eastern Europe [9,10]. Despite an estimated prevalence of 900,000 to 1.3 million new cases occurring annually, 20,000 to 30,000 deaths, and 350 million people at risk of infection, Leishmaniasis is still considered one of the most neglected diseases [9].

Leishmaniasis frequently occurs subclinically in immunocompetent subjects [7]. Moreover, within the leishmaniasis disease spectrum, VL is well known to be at greater risk of reactivation in immunocompromised hosts, most notably in PLWHA and patients undergoing immunosuppressive regimens [11,12,13]. Indeed, such cases have been increasingly reported in the literature lately, although none of them was found to be associated with IL-17 inhibitors [11].

Our following case report consists of the first described VL reactivation anti-IL-17 secukinumab treatment for psoriatic arthritis.

## 2. Case Report

**Figure 1:**
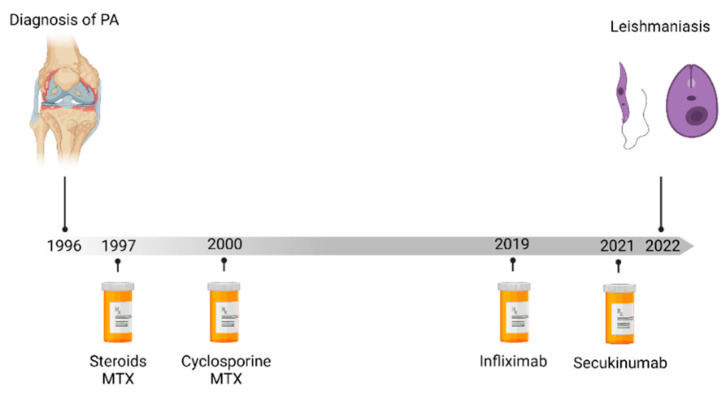
Timeline of treatment of psoriasis until diagnostic of visceral leishmaniasis. Abbreviations: MTX: metothrexate.

In March 2022, a 67-year-old Caucasian male diagnosed with psoriatic arthritis in 1996 was admitted to the Internal Medicine ward of our hospital in North-West Italy. His past rheumatologic history was characterized by steroids and disease-modifying antirheumatic (i.e., methotrexate and cyclosporine) drug failures. Moreover, in September 2021, anti-IL-17 secukinumab treatment was started with a significant clinical benefit due to the inefficacy of a two-year course of anti-TNF therapy with infliximab (Figure 1). However, from January 2022, secukinumab had to be prematurely discontinued due to a persistent fever, pancytopenia and a four-kilo weight loss.

Before starting secukinumab therapy between July and August 2021, he repeated a comprehensive microbiological screening with Mantoux test, QuantiFERON-TB Gold, HIV Ab, HCV Ab, HbsAg, HbsAb and HbcAg, which all yielded negative results. He also complained of arterial hypertension and was a former smoker in his past medical history. He was a retired office worker with a hobby of agriculture. He was living in the countryside with his wife, and he had had a dog two years previously that passed away in 2019 in unclear circumstances. Moreover, the patient had not travelled outside Italy in the previous five years.

At the time of admission, laboratory findings showed 8.2 g/dL haemoglobin, 53,000 platelets per μL, 1440 white cells per μL (respectively 620 neutrophils and 530 lymphocytes) and an IgG hypergammaglobulinemia (Table 1).

**Figure 2:**
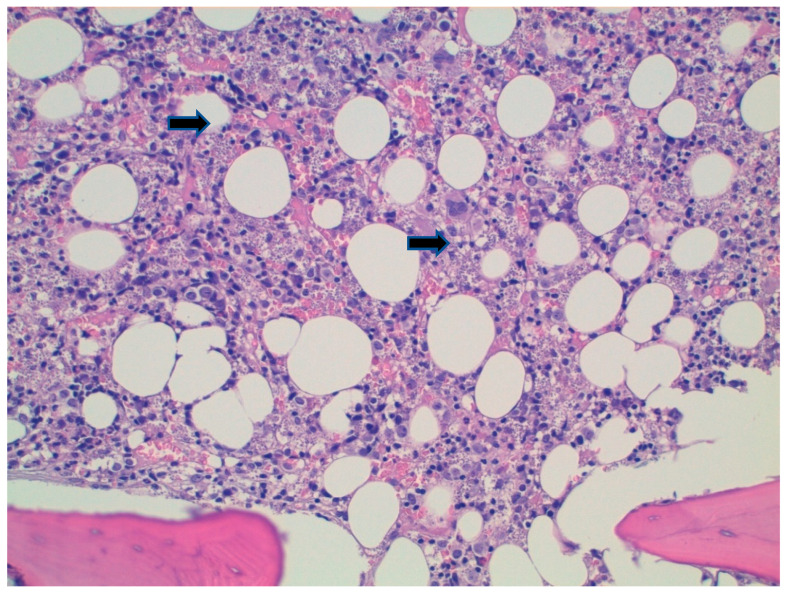
Hematoxylin-eosine stain on osteomedullary biopsy showing amastigotes (black arrows). **Caption:** Hematoxylin-eosin shows histiocytes containing innumerable hematoxylinophilic corpuscles compatible with Leishmania amastigotes (arrows), also present in the interstitium (20×).

A plain chest X-ray was negative. Echocardiography was performed, and found to be negative for endocarditic vegetations or pericardial involvement. Furthermore, a Total body Computed Tomography showed enlarged liver and concomitant splenomegaly of 20 cm. Due to persistent fever, further microbiological analysis was requested (Table 2), and in a positive serology, a Western Blot for *Leishmania* spp. was found. Due to the high suspicion of visceral leishmaniasis (VL), an osteomedullary bone biopsy was performed, showing multiple amastigotes (Figure 2), and a confident diagnosis of VL was made.

The patient started therapy with Liposomal Amphotericin B (L-AmB, 3 mg/Kg on days 1–5, followed by a dose on days 10, 17, 24, 31 and 38) with an immunocompromised patient regimen. The treatment was well tolerated, and laboratory and clinical values returned to normal. A second osteomedullary bone biopsy was performed which showed normal cellularity. However, since the patient presented with an exacerbation of pre-existent arthropathic psoriasis in May, secukinumab was re-started four weeks after the end of the L-AmB regimen without complications, and currently, the patient has reached the third month after the start of anti-IL17, with no clinical or laboratory signs of reactivation.

## 3. Discussion

VL is usually characterized by prolonged fever, weight loss, splenomegaly and hepatomegaly, progressive anemia or pancytopenia and hypergammaglobulinemia [7]. Moreover, the reactivation of latent *Leishmania* infection in chronic diseases and immunocompromised hosts is a broad and heterogeneous field in medicine and infectious diseases [11,12,13]. In particular, among patients with psoriasis, with or without psoriatic arthritis, reported cases of *Leishmania* infections and reactivations are growing in the literature [12]. 

Interestingly, the link between psoriasis and *Leishmania* spp. has historically been complicated and yet to be fully understood. On the one hand, some authors have argued that psoriasis might represent a protective factor against *Leishmania* infection [6]. On the other, a first-generation polyvalent vaccine manufactured with proteins from several cultured *Leishmania* species has proved effective in treating psoriasis [5].

Recently, Colomba et al. reported a brief literature review of patients with psoriasis (and psoriatic arthritis) and leishmaniasis [12]. Moreover, their literature review, which included cases from 2004 to 2020, showed that no patients undergoing immunosuppressive treatment with anti-interleukin-17 were reported [12].

We describe the first case of *Leishmania* reactivation in a patient who underwent secukinumab treatment.

Interleukin-17 (IL-17) inhibitors (i.e., ixekizumab, secukinumab and brodalumab) are used successfully in the biological treatment of psoriasis and psoriatic arthritis, but at the same time mediate the immune response against bacteria and fungi [14]. IL-17 is a well-studied cytokine and an essential player in the mammalian immune system. IL-17 was initially thought to be produced by CD4 T Helper 17 (Th17), but is now known to be produced by various other cells also, including macrophages, dendritic cells, CD8 Tc17, lymphoid tissue inducer, and γδ-T [15]. Although this cytokine plays an essential role in many infectious diseases, it promotes inflammatory pathology in autoimmunity and in other settings [16].

Previous research has shown that a Th1 immune response is protective against cutaneous variants of the disease, while a Th1/Th2 response is more typically reported against visceral infections [4]. The pro-inflammatory IL-17 cytokine family plays a significant role in the elimination of intracellular parasites in addition to the Th1/Th2 cytokine families. IL-17, which is produced by Th17 cells, is demonstrated to worsen illness in *Leishmania*-induced skin lesions [4,5]. This finding suggests a role for IL-17 in the pathophysiology of this condition [5]. Growth of IL-17 generating cells during vaccine-induced immunity, however, suggests a protective function for IL-17 [6]. Further evidence that IL-17 is involved in vaccine induced protective immunity comes from studies on humans with visceral leishmaniasis (VL), where it was found that IL-17 and IL-22 were linked to protection against re-exposure to *Leishmania* [4,5,6].

Furthermore, our patient underwent methotrexate, cyclosporine and subsequently infliximab for a long time, despite no clinical or laboratory signs of *Leishmania* spp. reactivation. According to Colomba et al., in 22 cases collected between 2004 and 2020, 14 and 9 patients had had ongoing treatment with methotrexate or infliximab, respectively [12].

Anti-TNF treatment-related immunosuppression seems to have a wider spectrum of interactions in the cytokine cascade than anti-IL-17 regimens, and also involves T helper cells (Th17) [15,16,17]. Recently, the functional role of IL-17-producing Th17 in psoriasis has been suggested by its reduction during successful anti-TNF treatment [15,16,17].

Moreover, Maritati et al. found a higher prevalence of subclinical leishmaniasis in patients with inflammatory rheumatic diseases receiving biological drugs than in those treated with other immunosuppressive drugs [18]. In addition, Kurizky et al. conducted a cross-sectional screening study in an area endemic to leishmaniasis with a cohort of 311 patients undergoing treatment with immune suppressive regimens [19]. They found that a total of 29 patients (seven serology alone, thirteen conventional PCR and nine real-time PCR) were positive for *Leishmania* spp. screening, but no patients using IL-17A inhibitors tested positive for *Leishmania* spp. [19].

Regarding previous cases reported of VL in psoriatic patients, middle-aged men with arthritic involvement were the most represented in the literature, and these clinical characteristics were also present in our patient [12].

Recently, Kurizky and colleagues published a systematic review that tried to assess the real burden of Leishmaniasis in the whole immunosuppressed population [19]. In their work, the mean age at the time of leishmaniasis development was 51.63 years and the mean duration of immunosuppression was 5.75 years [19].

They observed how the problem of patients treated with immunosuppressive drugs is mostly reported in European countries around the Mediterranean Sea. In Italy, the most common diseases are systemic lupus erytematous, rheumatic arthritis, Wegener disease and psoriatic arthritis, while the most frequent species of *Leishmania* are *L. infantum* and *L. donovani* [19].

Their systematic review showed unexpected results in the geographical distribution of the included cases. The countries that most often reported leishmaniasis in patients treated with immunosuppressive drugs were considerably different from the global picture of this disease in the general population. Normally, Leishmaniasis in Europe accounts for only 2% of all leishmaniasis cases in the world, but they reported that most of the cases of leishmaniasis in immunosuppressed individuals were in Europe (almost 80%) [20]. Chronic rheumatic conditions are more common in developed countries than in developing countries [21], and, for that reason, European countries around the Mediterranean coast have more access to modern immunosuppressive treatments than countries of South America and the Middle East, in which leishmaniasis is endemic [20].

The diagnosis of the reactivation of VL in our study, rather than acute infection, is related to different factors. First, the patient had contact with dogs and the countryside; moreover, his dog died precociously three years before diagnosis. Second, the patient lived in an area in which Leishmania is endemic. Third, splenomegaly and cytopenia showed a subacute or chronic infection that resembled a long-lasting or reactivated infection. In *Leishmania* spp. infections, past medical history, habits and hobbies, contact with animals and living in endemic countries may increase the risk of infection and reactivation under certain circumstances [21]. Fourth, the suspected reactivation of Leishmania could be due to infliximab or immunosuppressive agents that the patient took for many years before secukinumab.

Our patient’s response to L-AmB treatment was evaluated with clinical and laboratory follow-up, completed by an osteomedullary biopsy that turned negative six weeks after starting treatment. Several direct and indirect biomarkers for *Leishmania* spp. response after treatment have been proposed in the past, including PCR for *Leishmania,* which was unfortunately negative from admission [22].

Treatment restart with secukinumab for exacerbation of psoriatic arthritis was discussed with a rheumatologist and a senior infectious disease consultant. The patient was followed as an outpatient in both clinics with monthly follow-up through clinical and laboratory markers (including PCR for *Leishmania* spp.) that at the time of this report have steadily remained within normal limits.

## 4. Conclusions

Increasing awareness of *Leishmania* reactivation risk in endemic countries was testified by case reports and case series available in the literature, although a more standardized approach for screening populations at risk has yet to be proposed to reduce the risk of premature suspension of treatment and high mortality if left untreated for VL. Furthermore, of interest is the role of IL-17 in the elimination of intracellular parasites of *Leishmania* and in the pathways of immune-response against this microorganism.

## Figures and Tables

**Table 1:** Timeline from admission to first 3-month follow-up.

	Day 0	Day 5	Day 10	Day 38	1 Month PT	3 Months PT
Total WBC count (10^3^/uL)	1.44	2.35	3.0	3.27	6.07	5.40
Lymphocytes (absolute)	530	770	770	940	1370	1550
Neutrophils (absolute)	620	1.220	1710	1880	3990	3220
Hb (g/dL)	8.2	8.4	8.5	9.6	11.0	11.8
PLTS (10^3^/uL)	53	98	134	197	191	161
LDH (U/L)	351	NA	NA	NA	NA	NA
IgG (g/L)	24	NA	NA	21	22.6	NA
Albumin (g/L)	26	NA	NA	NA	NA	NA
Ferritin (ng/mL)	1075	NA	NA	NA	NA	NA
GOT	19	24	14	16	NA	12
GPT	20	29	9	13	NA	8
Bilirubin Total (mg/dL)	0.9	NA	0.6	NA	NA	NA
Creatinine (mg/dL)	1.45	2.5	1.65	1.42	NA	1.4
Protein C-reactive (mg/dL)	93.3	15.7	29.7	43.5	33.5	10.7
Procalcitonin (ng/mL)	0.8	0.18	0.08	NA	0.10	0.04
Protrombin Time	66%	NA	NA	NA	NA	NA

Abbreviations: PT: post-treatment; WBC: White Blood Cell; Hb: Hemoglobin; PLTS: platelets; LDH: lactate dehydrogenase; GOT: glutamic-oxaloacetic transaminase; GPT: glutamic-pyruvic transaminase; NA: not available.

**Table 2:** Microbiological Screening for Pancytopenia and Fever.

Microbiological Screening on Admission	
HIV ½ Ab and p24	*Negative*
HBsAg, antiHbs, anti-HBc	*Negative*
HCV Ab	*Negative*
Treponema total Ab	*Negative*
CMV	*IgM negative*, *IgG positive*
EBV	*VCA IgG*, *Positive*, *EBNA IgG positive*
HSV 1 & 2	*IgM negative*, *IgG positive*
HHV-6	*IgM negative*, *IgG positive*
Adenovirus	*IgM negative*, *IgG negative*
Parvovirus B19	*IgM negative*, *IgG negative*
*Mycobacterium tuberculosis* Quantiferon	*Negative*
Galactomannan Ag	*Negative*
Beta D-glucan Ag	*Negative*
*Leishmania* spp.	*WB p14 positive*, *p16 positive*. *PCR: negative*
COVID-19	*Negative*

Abbreviations: HIV: human immunodeficiency virus; HB: hepatitis B; HCV: hepatitis C virus; CMV: cytomegalovirus; EBV: Epstein–Barr virus; HSV: herpes virus; HHV-6: human herpes virus.

## Data Availability

Not applicable.
